# Effects of a short message service (SMS) by cellular phone to improve compliance with fasting guidelines in patients undergoing elective surgery: a retrospective observational study

**DOI:** 10.1186/s12913-020-06039-y

**Published:** 2021-01-06

**Authors:** Faizan Zia, Luka Cosic, Angela Wong, Adam Levin, Patrick Lu, Craig Mitchell, Michael Shaw, Fred Rosewarne, Laurence Weinberg

**Affiliations:** 1 Department of Anesthesia, Ballarat Base Hospital, Ballarat, VIC 3350 Australia; 2 Department of Anesthesia, Austin Hospital, 145 Studley Road, Heidelberg, VIC 3084 Australia; 3 Department of Anesthesia, Royal Melbourne Hospital, Parkville, VIC 3050 Australia; 4 The Univesity of Melbourne, Department of Surgery,Austin Health, Melbourne, VIC 3084 Australia

**Keywords:** Fasting, Anesthesia, Peri-operative, Pre-operative

## Abstract

**Background:**

Contemporary perioperative fasting guidelines aim to alleviate patient discomfort before surgery and enhance postoperative recovery whilst seeking to reduce the risk of pulmonary aspiration during anesthesia. The impact of a short message service (SMS) reminder on fasting guideline compliance is unknown. Therefore, we performed a retrospective observational study and quality improvement project aiming to quantify the extent of excessive and prolonged fasting, and then assessed the impact of a SMS reminder in reducing fasting times.

**Methods:**

After ethics committee approval we performed a retrospective observational study investigating preoperative fasting times of adult patients undergoing elective surgery. First, we assessed whether the fasting guideline times were adhered to (Standard Care group). All patients received internationally recommended fasting guidelines in the form of a written hospital policy document. We then implemented an additional prompt via a mobile phone SMS 1 day prior to surgery containing a reminder of fasting guideline times (SMS group). The primary aims were to compare fasting times between the Standard Care group and the SMS group.

**Results:**

The fasting times of 160 patients in the Standard Care group and 110 patients in the SMS group were evaluated. Adherence to the fasting guidelines for solids occurred in 14 patients (8.8%) in the Standard Care group vs. Twenty-two patients (13.6%) in the SMS group (*p*=0.01). Adherence to the fasting guidelines for fluids occurred in 4 patients (2.5%) in the Standard Care group vs. Ten patients (6.3%) in the SMS group (*p*=0.023). Patients in the Standard Care group had a longer median (inter-quartile range (IQR)) fasting time for fluids compared the SMS group [6.5 h (IQR 4.5:11) vs 3.5 h (IQR 3:8.5), *p*< 0.0001]. Median fasting times for solids were 11 h (IQR 7:14) in the Standard Care group and 11.5 h (IQR 7:13.5) in the SMS group (*p*=0.756).

**Conclusion:**

Adherence to internationally recommended fasting guidelines for patients undergoing elective surgery is poor. The introduction of a fasting guideline reminder via a mobile phone SMS in addition to a written hospital policy improved adherence to fasting advice and reduced the fasting times for fluids but not for solids. The use of an SMS reminder of fasting guidelines is a simple, feasible, low-cost, and effective tool in minimising excessive fasting for fluids among elective surgical patients.

**Trial registration:**

ACTRN12619001232123 (Australia New Zealand Clinical Trials Registry). Registered 6th September 2019 (retrospectively registered).

## Background

Pulmonary aspiration of gastric contents carries significant morbidity and mortality [1]. Accordingly, perioperative fasting guidelines exist aiming to reduce the risk of aspiration during anesthesia and limit the severity of sequelae following aspiration should it occur. The rationale for fasting prior to elective surgery is to reduce the gastric acidity, particulate matter and volume of gastric contents [2, 3].

Historically the first documented *nulla* per os (NPO) instructions were outlined in 1855 when Dr. John Snow gave direction to avoid eating a meal prior to inhalation of chloroform to limit the occurrence of vomiting [4]. As early as the 1860s there are documented reports of authors recommending fasting for solids for 6 h and for fluids from between 30 min to 3 h prior to surgery [4]. Interestingly, these directives conform to contemporary fasting guidelines, which aim to alleviate patient thirst, hunger, anxiety, and discomfort before surgery, and also seek to enhance postoperative recovery after surgery [3]. However, in 1946 Mendelson demonstrated the risks associated with aspiration of gastric contents, leading to the recommendation of NPO from midnight and the widespread belief that longer fasting times reduced aspiration risk [5]. Despite new recommendations in modern anesthesia and general consensus amongst anesthesia organisations around the world regarding optimal fasting times for both solids and fluids [6–11], the dogma of NPO from midnight has been difficult to overcome and there are still occasions where patients are receiving these fasting instructions today contrary to current recommendations [12].

Contemporary fasting guidelines are based largely on expert opinion and with reference to known gastric physiology, and there is limited evidence that strict adherence to recommended fasting guidelines improves patient outcomes after elective surgery. Although prolonged fasting has often been considered innocuous, there is emerging evidence suggesting prolonged fasting may worsen outcomes without necessarily reducing the risks of anesthesia [13–20]. Potential harms include increased risk of dehydration, unstable blood sugar levels, increased gastric acidity, discomfort, and increased incidence of postoperative nausea and vomiting [13–18, 20]. Given the growing body of evidence concerning the harms of prolonged fasting, it is important that fasting times be minimised, and guidelines be adhered to. In response to the potential harms and lack of clear benefit, there has been a recent call to change guidelines to reduce fasting times for fluids to 1-h, in line with recent changes to guidelines for children [3].

Despite hospitals having well established and consistent fasting guidelines, patients undergoing elective surgery are frequently fasted for longer than recommended by fasting guidelines [3, 20–24]. Corroborating these findings, anesthesiologists at our institution anecdotally noted that patients were fasting excessively and often unnecessarily. To support these observations, we performed a retrospective observational study to determine the extent of prolonged and excessive fasting amongst elective surgery patients. We aimed to assess patients’ adherence to standard fasting guidelines. Seeking to improve adherence to fasting guidelines, we then investigated the impact that an additional mobile phone short message service (SMS) sent to patients the day before surgery would have on fasting times. Based on evidence supporting the use of SMS reminders in adherence to bowel preparation prior to endoscopy, vaccines and outpatient appointments [25–33], we hypothesised that the usage of an SMS reminder 1 day prior to surgery would assist patients in adhering to fasting guidelines and thus reduce excessive fasting.

## Methods

First, we assessed the fasting times amongst elective surgery patients at our institution. Data was collected between February 3rd to the 13th, 2014. Inclusion criteria were adult patients (> 18 years) undergoing elective surgery with a day of surgery admission. All surgical specialties and all procedures were considered, as were morning and afternoon surgeries. All patients were required to have a mobile phone number documented on their medical record. Exclusion criteria were emergency procedures, out of hours procedures, morbid obesity (body mass index > 35 kg/m^2^), pediatric patients, patients with previous gastric or bypass surgery, pregnancy, and any patient with a history of delayed gastric emptying e.g. achalasia. We also excluded any inpatient awaiting non-time critical surgery due to the variability of fasting advice given to these patients by medical and nursing staff.

All patients in this cohort received standard fasting guidelines according to our hospital policy (Standard Care group). This included verbal instructions at the time of surgical and/or anesthesia clinic which occurred within 2 weeks of surgery, an informative colour brochure in the English language sent to the patient at the time of surgery booking (1 week prior to surgery), and then a reminder phone call by a dedicated surgical nurse 1 day prior to surgery. The fasting protocol used at our institution was in accordance with The Australian and New Zealand College of Anesthesiologists (ANZCA) guidelines of 6 h for solids and 2 h for < 200 ml of clear fluids [3], with the exception of morning list patients, who were advised to fast for solids from midnight (2.5 h more than recommended). Fasting times for the Standard Care group were obtained from preoperative day stay unit records that were completed at the time of admission. On admission every patient undergoing surgery at our institution was asked about their fasting status for both solids and fluids as part of standard care. This information was entered into the patient medical records.

Following this initial audit, we then initiated a quality improvement program for patients awaiting elective surgery (SMS group). Similar to the inclusion criteria for the audit, we included adult patients (> 18 years) undergoing elective surgery with a day of surgery admission. We excluded patients undergoing emergency procedures, morbid obesity (body mass index > 35 kg/m^2^), patients with previous gastric or bypass surgery, pregnancy, history of delayed gastric emptying, and all inpatients. Data for the SMS group was collected from the April 28th to May 13th, 2014 during which we implemented an additional prompt 1 day prior to surgery via a mobile phone SMS containing a reminder of fasting times. All patients also receiving standard fasting guidelines instructions as per the standard hospital policy i.e. verbal instructions at the time of their outpatient clinic, colour brochure sent 1 week prior to surgery, and reminder phone call 1 day prior to surgery. The information provided by each method of communication is outlined in Table 1*.*

**Table 1:** Information provided to patients during four points of contact

**Verbal instructions at outpatient clinic**	
Morning list patients - Fast from midnight for solids - Fast from 06:30 for clear fluids Afternoon list patients - Fast from 07:30 for solids - Fast from 11:30 for clear fluids Both groups received verbal information on reasons for fasting, aspiration risk, and details of what constituted solids and clear fluids, as outlined in the methods.	
**Written instructions in mail (color brochure)**	
Morning list patients - Fast from midnight for solids - Fast from 06:30 for clear fluids Afternoon list patients - Fast from 07:30 for solids - Fast from 11:30 for clear fluids Both groups received written information on reasons for fasting, aspiration risk, and details of what constituted solids and clear fluids, as outlined in the methods.	
**Verbal instructions by telephone 1 day prior to surgery**	
Morning list patients - Fast from midnight for solids - Fast from 06:30 for clear fluids. Afternoon list patients - Fast from 07:30 for solids - Fast from 11:30 for clear fluids Both groups received verbal information on reasons for fasting, aspiration risk, and details of what constituted solids and clear fluids, as outlined in the methods.	
**SMS reminder**	
Morning list patients - Fast from midnight for solids - Fast from 06:30 for clear fluids Afternoon list patients - Fast from 07:30 for solids - Fast from 11:30 for clear fluids	

The SMS was sent to the patient’s registered mobile phone by a dedicated perioperative staff member who had confirmed the theatre list for the following day prior. The SMS was in the English language, clearly articulating the fasting times for both solids and fluids. Solids were described as a “very light meal e.g. cereal or toast”. Clear fluids were described “water, clear cordial or apple juice, or black tea or black coffee with NO milk”. The SMS messages were sent by staff of the pre-operative day stay unit who were aware of the study protocol.

When the patient arrived for their scheduled surgery, they were asked to complete a short questionnaire in the day of surgery waiting area. The following questions were asked:

1. What time did you last have something to eat?
2. What time did you last have something to drink?
3. Do you remember receiving the fasting guidelines brochure with your booking letter?
4. Did you receive fasting guidelines via a telephone call the day before surgery?
5. Did you receive fasting guidelines via a SMS message the day before surgery?

Fasting time for solids was calculated from the last time the patient ate until the start of the morning list at 08:30 or until the start of the afternoon list at 13:30, depending on which surgical list the patient had been booked for. Similarly, fasting time for clear fluids was calculated from the last time the patient drank clear fluids until the start of the morning or afternoon list. We received ethics exemption on the grounds of being a quality improvement project from the Ballarat Health Services and St John of God Hospital Ballarat Human Research Ethics Committee for both parts of this study (HREC/12/BHSSJOG/76).

### Statistical analysis

A statistical software package (SPSS Version 19.0; IBM Co, Armonk, NY, USA) was used for statistical analysis, with a two-tailed *P* value less than 0.05 as statistically significant. Results were expressed as either a median (inter-quartile range (IQR)) or in the form of frequencies unless otherwise stated. Comparisons between categorical variables were determined by chi-square and Fisher’s exact test as appropriate. Non-categorical variables were assessed by the Mann-Whitney U test.

## Results

There were 270 patients who underwent elective surgery over the study period. There were 160 patients in the Standard Care group and 110 patients in the SMS group. When categorising by surgical specialty, the Standard Care group had 54 (34%) general surgery patients, 22 (14%) gynecology patients, 36 (22%) orthopedics patients, 21 (13%) urology patients, 20 (13%) ear nose and throat surgery patients, 5 (3%) ophthalmology patients and 2 (1%) vascular patients. The SMS group contained 24 (22%) general surgery patients, 31 (28%) gynecology 31 patients, 22 (20%) orthopedics patients, 15 (14%) urology patients, 5 (4.5%) ear nose and throat surgery patients, 8 (7%) ophthalmology patients, and 5 (4.5%) vascular patients.

The median age was 58 years (IQR 41:67) and 59 years (IQR 35:70) for the Standard Care and SMS groups respectively. Adherence to the fasting guidelines for solids occurred in 14 patients (8.8%) in the Standard Care group vs. 22 patients (13.6%) in the SMS group (*p*=0.01). Adherence to the fasting guidelines for fluids occurred in 4 patients (2.5%) in the Standard Care group vs. 10 patients (6.3%) in the SMS group (*p*=0.023). Patients in the Standard Care group had a median fasting time of 6.5 h (IQR 4.5:11) compared to 3.5 h (IQR 3:8.5) in the SMS group; *p* < 0.0001). Median fasting times for solids were 11 h (IQR 7:14) in the Standard Care group and 11.5 h (IQR 7:13.5) in the SMS group (*p*=0.756). Median fasting times for fluids were significantly lower for the SMS group than the Standard Care group (3.5 h (IQR 3:8.5) vs 6.5 h (IQR 4.5:11), *p*< 0.0001). Only 8.8% of patients in the Standard Care group fasted according to guidelines for solids, with 2.5% fasting appropriately for fluids. Only one patient in the Standard Care group fasted for an insufficient period of time for solids and fluids, however still proceeded to have surgery. In the SMS group 13.6% of patients fasted appropriately for solids and 6.3% for fluids, with only one patient fasting for an insufficient period of time for solids.

Of the 110 patients in the SMS group, 37 (33.6%) patients reported receiving a brochure advising them of fasting guidelines. 92 (83.6%) patients reported receiving a phone call prior to surgery with a reminder of fasting guidelines, and 85 (77.2%) patients reported receiving the SMS message containing fasting guidelines. Subgroup analysis of compliance to fasting guidelines between the morning and afternoon lists are presented in Table 2. There were no reported pulmonary aspirations in either group of patients.

**Table 2:** Subgroup analysis of compliance to fasting guidelines presented as median (IQR)

AM List fasting time (hours)				PM List fasting time (hours)			
Clear fluids		Solids		Clear fluids		Solids	
**Standard care**	**SMS**	**Standard care**	**SMS**	**Standard care**	**SMS**	**Standard care**	**SMS**
9.5 (3.6:11.5)	3.5 (2.5:11.1)	11.5 (10.1:13.5)	12.2 (10:13.5)	6.2 (4.5:7)	4 (3:6)	7.2 (6.5:16.5)	6.5 (6:15.3)
*P* = 0.008		*P* = 0.394		*P* < 0.0001		*P* = 0.105	

## Discussion

We performed a retrospective observational study followed by a prospective quality improvement initiative aiming to quantify the extent of excessive and prolonged fasting, and then assess the impact of a short message service reminder in reducing fasting times. Notably, we found that over 90% of elective surgery patients fasted excessively at our institution for both solids and fluids, corroborating anecdotal observations by clinical staff. We found that using an SMS reminder of fasting guidelines improved adherence to fasting guidelines and reduced the fasting times for fluids, however contrary to our hypothesis, had little to no effect on fasting times for solids. Given that adherence to internationally recommended fasting guidelines for patients undergoing elective surgery is poor, the use of an SMS reminder of fasting guidelines can be used as a simple, feasible, low-cost tool in minimising excessive fasting for fluids among elective surgical patients remains.

Interestingly, patients on the morning list were less compliant with fasting guidelines for both solids and fluids. We postulate that this was most likely due to an unwillingness to eat late at night amongst patients, and similarly an unwillingness to wake early in the morning to rehydrate. Patients at present may lack the motivation to do so in part due to the information that is conveyed to them. Fasting information often focusses on the risks of aspiration and necessity of fasting, without effectively iterating the importance of ensuring patients maintain fluids and continue to consume carbohydrate rich fluids as is recommended in enhanced recovery after surgery protocols. By providing patients information regarding reducing fasting times to improve perioperative outcomes, clinicians can reinforce the need to limit fasting and encouraging patients to eat at 6 h preoperatively and drink fluids up to 2 h preoperatively, which may in turn improve adherence.

In addition, morning list patients were advised to fast for 2.5 h more than afternoon list patients (i.e. from midnight), which given the discrepancy in fasting times between morning and afternoon lists, may not continue to be best practice. We found that less than 10% of elective surgical patients fasted for the guideline recommended duration for solids, and only 2.5% fasted appropriately for fluids. This improved with the use of an SMS reminder, increasing adherence to guidelines for solids to approximately 13% and fluids to a little over 6%, although the increase was only statistically significant for fluids.

Our findings support the utilisation of an SMS reminder for reducing excessive fasting. Whilst the SMS alone was not able to limit fluid fasting times to those recommended by best-practice guidelines, the improvement demonstrated renders SMS notifications as a useful tool in preoperative care. Although our findings were statistically significant, the improvement in fasting adherence in absolute terms was modest, yet we believe them to be clinically significant. It is unlikely that any single intervention will lead to large improvements in fasting adherence, although as with much of perioperative medicine in the modern era, clinically significant improvements are achieved through the aggregate of marginal gains. SMS reminders can serve as one such aggregate and given the low cost and easy implementation with many institutions already using SMS for appointment reminders, it remains an actionable tool as one of many steps to improve fasting adherence.

Our findings also suggest that there are improvements to be made in preoperative care and optimisation of patients in our institution. Given that enhanced recovery after surgery programmes and extensive prehabilitation are being utilised to improve patient outcomes in our hospital, additional focus on optimising patient fasting times is a simple and low-cost strategy to further improve patient outcomes. Given the low-cost of the SMS, and the ease of implementation, our findings support the implementation of an SMS reminder for fasting times in other institutions, where fasting times are discordant with current international recommendations. This is particularly pertinent given that a number of studies have demonstrated improved patient outcomes associated with reduced fluid fasting times [13, 14]. Although the use of an SMS reminder in improving adherence to fasting guidelines has not been investigated previously, there is an abundance of contemporary literature demonstrating improvement in adherence to bowel preparation prior to endoscopy, to vaccination schedules, and outpatient appointments with the use of SMS reminders [25–33]. These findings across other areas of healthcare are consistent with our findings and support the implementation of this low-cost tool in improving healthcare outcomes.

Our study highlights a number of potential targets for improving preoperative fasting times. Chiefly, we found that only one third of patients reported receiving a paper brochure of fasting guidelines. We postulate that this limited recollection amongst patients is primarily due to the large quantity of information given to patients during their preadmission visit, along with the length of time elapsing between the preadmission visit and the surgery. However, it is also likely that the information provided to patients may not have been sufficient to alter pre-existing knowledge or beliefs regarding fasting. The traditional dogma of *nulla* per os with regards to fasting lingers to this day despite updated fasting guidelines, and both clinicians and patients may be affected by this. This can be improved by reinforcing the correct fasting guidelines on multiple occasions, via phone call and additionally SMS, which additionally also resulted in a greater proportion of patients reported being able to recollect having received information on fasting guidelines. This finding suggests the use of multiple prompts is superior in improving patient recollection and further supports the use of an SMS reminder for improving adherence to fasting guidelines.

Our audit has a number of key limitations. Although our data was collected 5 years ago, it still reflects the importance of the anesthesia fasting instructions undertaken in many institutions. Fasting times for patients were calculated from the time patients last consumed solids or fluids to the beginning of the morning or afternoon lists. Whilst this allowed for flexibility of surgical scheduling in the event of unforeseen circumstances, it limited the precise fasting times being documented. Accordingly, fasting times for patients scheduled for later on the operating lists may be underestimated, although this would further support our finding of excessive fasting times. We acknowledge the discrepancy in fasting advice given to patients on the morning list compared with those on the afternoon list. Patients on the morning list were asked to fast from midnight, or 2.5 h longer than those on the afternoon list. This advice was well-intentioned, seeking to minimise disruption to patient’s routines, however it also likely impacted on the longer fasting times observed amongst morning list patients.

Additionally, our methods of providing informed fasting instructions to patients i.e. verbally in the preadmission surgical/anesthesia clinic, information brochure and preoperative phone call; may not be generalizable to all hospitals, although are an accepted standard across most similar institutions. Whilst patients in the SMS group were asked about their recollection of receiving fasting advice, this was not done for Standard Care group. Comparisons regarding recall are therefore not possible. This is unlikely to have affected our results, however it does remove the possibility of comparison between groups and limits our ability to comment on whether patient recollection of fasting guidelines may contribute to excessive fasting. Importantly the preoperative phone call and additional SMS were only conveyed to patients in the English language, which may help to account for why almost approximately 1 in 5 patients did not recall receiving the phone call or the SMS.

Our study did not consider whether the phone number to which the SMS reminder was sent belonged to the patient, or whether it belonged to a caregiver or family member. This may have led to a portion of the cohort not receiving the SMS, which may have resulted in an underestimate of the impact of SMS reminder on improving fasting adherence and underestimated recall. This is more likely to reflect a real-world scenario where many patients will likely rely on caregivers and family members to both receive SMS notifications and translate the necessary information for them. Finally, this study did not assess the effects of prolonged fasting times on patient’s satisfaction or other important clinical outcomes.

Our findings support existing literature such as the BIGFAST multicentre study, which found that 88% of patients fasted more than 6 h for both solids and fluids in a study of 3715 Brazilian patients [23]. Additionally, in an audit of 45 patients, Nagaratnam et al.^22^ reached a similar conclusion, reporting that 92% of patients fasted more than recommended times for solids, and 95% of patients fasting more than recommended fasting times for fluids. Further, our findings are consistent with previous publications which have reported that most patients who undergo surgery fail to recall a large amount of the information presented to them [34, 35]. A large body of literature demonstrating low patient recall of preoperative instructions for informed consent after major surgery exists [36], and this raises the possibility that by the day of surgery participants may have forgotten the fasting instructions previously explained. These findings draw into consideration the effectiveness of our current methods in providing patients with information about fasting.

At our institution all patients had contact with a number of healthcare professionals prior to their scheduled surgery and were sent an information brochure with fasting instructions 1–2 weeks prior to surgery. There were therefore multiple opportunities for clinicians to reinforce fasting information and to check whether fasting instructions were retained and understood. It is possible that patients were given information far beyond the number of new items human beings can remember. This may have contributed to the lack of recall of the phone call or SMS received. Patients do not always recall what they were told, and what they do remember is not always what the clinician believes to be the most important information [34]. Ultimately, patient recollection is inherently embedded in a complex process where clinicians must try to understand how information is conceptualized and retained and seek to deliver information in a manner which patients will recall.

Our findings indicate that there is a role for SMS reminders in improving fasting adherence, although further studies are needed. Our study demonstrated only modest improvements to fasting adherence. Part of this may be due to the information provided to patients. Future studies should address the information provided to patients regarding fasting, how best to frame the positive impacts of reduced fasting times, and how best to convey that information through an SMS reminder. Future studies could address whether or not it may be beneficial to send a SMS reminder just prior to commencing fasting to notify patients to eat a meal or drink a carbohydrate rich drink. This could potentially result in an improvement in adherence to fasting guidelines.

## Conclusion

An SMS reminder of fasting guidelines sent to patient’s mobile phones improved adherence to recommended fasting times, however overall adherence to these internationally recommended fasting guidelines for patients undergoing elective surgery was poor. Further the SMS reminder achieved a significant reduction in the fasting times for fluids but not for solids. The use of an SMS reminder of fasting guidelines is a simple, low-cost and effective tool in minimising excessive fasting for fluids among elective surgical patients.

## Data Availability

The datasets used and/or analysed during the current study are available from the corresponding author on reasonable request.

## Abbreviations

SMS: Short message service
IQR: Inter-quartile range
